# Potential for Biological Control of *Pythium schmitthenneri* Root Rot Disease of Olive Trees (*Olea europaea* L.) by Antagonistic Bacteria

**DOI:** 10.3390/microorganisms10081635

**Published:** 2022-08-12

**Authors:** Ikram Legrifi, Jamila Al Figuigui, Hajar El Hamss, Abderrahim Lazraq, Zineb Belabess, Abdessalem Tahiri, Said Amiri, Essaid Ait Barka, Rachid Lahlali

**Affiliations:** 1 Phytopathology Unit, Department of Plant Protection, Ecole Nationale d’Agriculture de Meknès, Km 10, Rte Haj Kaddour, BP S/40, Meknès 50001, Morocco; 2 Laboratory of Functional Ecology and Environmental Engineering, Sidi Mohamed Ben Abdellah University, P.O. Box 2202, Route d’Imouzzer, Fez 30000, Morocco; 3 Plant Protection Laboratory, Regional Center of Agricultural Research of Oujda, National Institute of Agricultural Research, Avenue Mohamed VI, BP428 Oujda, Oujda 60000, Morocco; 4 Unité de Recherche Résistance Induite et Bio-Protection des Plantes-EA 4707, Université de Reims Champagne-Ardenne, 51100 Reims, France

**Keywords:** *Pythium* *schmitthenneri*, antifungal effect, biocontrol, olive trees, root rot

## Abstract

Several diseases affect the productivity of olive trees, including root rot disease caused by *Pythium* genera. Chemical fungicides, which are often used to manage this disease, have harmful side effects on humans as well as environmental components. Biological management is a promising control approach that has shown its great potential as an efficient eco-friendly alternative to treating root rot diseases. In the present study, the antagonistic activity of ten bacterial isolates was tested both in vitro and in planta against *Pythium schmitthenneri*, the causal agent of olive root rot disease. These bacterial isolates belonging to the genera *Alcaligenes*, *Pantoea*, *Bacillus*, *Sphingobacterium*, and *Stenotrophomonas* were chosen for their potential antimicrobial effects against many pathogens. Results of the in vitro confrontation bioassay revealed a high reduction of mycelial growth exceeding 80%. The antifungal effect of the volatile organic compounds (VOCs) was observed for all the isolates, with mycelial inhibition rates ranging from 28.37 to 70.32%. Likewise, the bacterial cell-free filtrates showed important inhibition of the mycelial growth of the pathogen. Overall, their efficacy was substantially affected by the nature of the bacterial strains and their modes of action. A greenhouse test was then carried out to validate the in vitro results. Interestingly, two bacterial isolates, *Alcaligenes faecalis* ACBC1 and *Bacillus amyloliquefaciens* SF14, were the most successful in managing the disease. Our findings suggested that these two antagonistic bacterial isolates have promising potential as biocontrol agents of olive root rot disease.

## 1. Introduction

Olive trees (*Olea europeae* L.) were among the earliest cultivated fruit trees on the Mediterranean shores and are regarded as one of the most suitable crops for the Mediterranean climate [1]. Olive also plays a key socio-economic role in many countries including Morocco [2]. However, olive trees are susceptible to attacks by various soil-borne pathogens. These enemies decrease olive tree productivity since they affect both produced quantities and oil organoleptic quality [3,4].

Many pathogens such as *Fusarium solani*, *F. oxysporum*, *Rhizoctonia solani*, *Phytophthora* spp., and *Pythium* spp. have been linked to root rot diseases in both nurseries and new orchards where olives are grown worldwide [5,6,7,8,9,10,11,12]. *Pythium* species survive in the organic debris and soil as oospores, hyphae, and sporangia, which can persist for many years as oospores under unfavorable conditions [6]. In the absence of its host plants, the pathogen may live for many years and can become destructive when favorable conditions are present [13]. In most cases, symptoms of *Pythium* root rot are foliar yellowing, defoliation, chlorosis, and finally the death of infected plants [14,15,16,17]. Effective disease management is critically required.

Several agricultural practices, including crop rotation, soil solarization, and chemical use, have been adopted to control *Pythium* species [18]. Farmers’ primary method of reducing losses caused by these pathogens is, however, the use of fungicides [6]. The systemic fungicide “metalaxyl” has been quite effective in controlling *Pythium* and minimizing the disease incidence. Unfortunately, the effectiveness of fungicides can be limited in several ways. Under favorable conditions, they become progressively ineffective in controlling phytopathogens while causing serious environmental and human safety concerns [4]. Furthermore, these measures may lose their effectiveness in controlling the pathogen, as several reports have shown the emergence of *Pythium* spp. strains resistant to metalaxyl, metalaxyl-M, and Mefenoxam [19,20,21]. Therefore, it is crucial to develop alternative agricultural management practices.

Over the past two decades, biological control methods have been seen as promising, environmentally friendly, and sustainable alternatives to managing plant diseases [22]. These alternatives involve the use of biological control agents (BCAs), which decrease either the quantity of inoculum or the activity of pathogens [23,24]. These BCAs destroy their targets by a variety of mechanisms, including antibiosis, direct parasitism, competition for nutrition and space, and perhaps induced resistance [25,26,27]. The main advantage of applying a BCA is that it is extremely specific to a pathogen and so deemed harmless to non-target species [28].

The use of bacteria to control fungal diseases is a strategy that is part of sustainable and eco-friendly production [29]. Certain antagonistic biological control agents of the genera *Bacillus* spp. [30,31], *Pseudomonas* spp. [32,33], *Streptomyces* spp. [34,35], and *Trichoderma* spp. [36,37] have been highly effective in the management of pathogens causing soil-borne diseases such as *Fusarium* spp., *Rhizoctonia solani*, *Phytophthora* spp., and the *Pythium* genera. To date, only a few studies has been conducted on the adoption of antagonistic BCAs to control *Pythium* root rot on olive trees.

Ten bacterial strains, isolated from the citrus rhizosphere and blossoms of pear, apple, and quince trees, were recently chosen for their antagonistic abilities against a variety of pathogens [38,39,40]. The ability of these bacterial strains to produce enzymes such as amylase, cellulase, and protease was previously assessed. The principal aims of the current study were to (i) examine in vitro the efficacy of these bacterial strains in suppressing *P. schmitthenneri* mycelial growth, and (ii) evaluate their in vivo potential to control root rot disease on olive trees.

## 2. Materials and Methods

### 2.1. Fungal Preparation

*P. schmitthenneri* MZ466379, used in the present study, was isolated from symptomatic roots of olive trees during the 2020 growing season in Morocco and characterized as previously described [41]. Fungi colonies were subcultured from a 7-day culture on potato dextrose agar medium (PDA) [42] supplemented with an antibiotic (streptomycin sulfate at 50 g/mL) and incubated in the dark at 25 °C before experiments.

### 2.2. Bacterial Strains

The bacterial strains (10) tested in this work were originally isolated from apple, pear, quince trees, and citrus rhizosphere, identified and screened for their antagonistic activity against various pathogens [38,39,40], and are part of the collection of the Phytopathology Unit of ENA-Meknès. These bacterial strains were *Alcaligenes faecalis* (1 strain), *Pantoea agglomerans* (3 strains), *Bacillus amyloliquefaciens* (1 strain), *B. halotolerans* (1 strain), *B. subtilis* (1 strain), *B. xiamenensis* (1 strain), *Sphingobacterium multivorum* (1 strain), and *Stenotrophomonas maltophilia* (1 strain). Before performing the experiments, the bacterial cells were kept in an LB medium at 27 ± 1 °C for 24 h [43].

### 2.3. In Vitro Confrontation Bioassay

The capacity of the 10 selected bacteria to inhibit the hyphal growth of the pathogenic fungus was assessed using a dual culture test as described by Lahlali et al. [44]. A colony of 24-hour bacterial cultures and a 7-day pathogen culture were employed for this purpose. Each bacterium was streaked over a PDA medium in 4 equally spaced strips (3 to 4 cm in length) from the center of the Petri plate. Then, a 5 mm diameter mycelial disc of a fresh pathogen culture was deposited in the Petri plate center pre-seeded with bacterial isolates. To compare results, a negative control was used by placing the fungal culture disc in the center of Petri plates without any bacteria. The assay was conducted twice with four replicates for each pathogen/treatment combination across time. The diameter of mycelial growth was measured after 6 days of incubation at 25 °C, control conditions for the fungal colony to invade the entire plate. The inhibition rate was calculated using the formula described by Trivedi et al. [45]: Inhibition rate (%) = (diameter of fungal colony in control − diameter of fungal colony in treatment)/diameter of the fungal colony in control treatment × 100.

### 2.4. Effect of the Bacteria on the Cytology of Pythium

The influence of each antagonistic bacterium on the structure and morphology of the pathogenic mycelium was investigated. Microscopic observations were performed on 6-day-old culture Petri dishes using a light microscope (Ceti Microscopes NLCD-307B, Chalgrove, UK). A section of mycelium was taken from the fungal growth zone and deposited between slides for microscopic observation. Under the light microscope (40×), existing hyphal damage or cytological alterations, such as vacuolation, deformation, and hyphal swelling, generated by antagonistic bacteria were recorded in contrast to the control.

### 2.5. Volatile Organic Compounds (VOCs) Bioassay

The test was carried out to evaluate the ability of the bacteria used to inhibit fungal growth at a distance, which indicates the emission of VOCs by these antagonistic bacteria [46]. On LB medium, the bacteria were cultivated in three streaks and incubated at 28 °C. After 24 h, the Petri-plate lid of the ten cultures was removed and replaced by the bottom of another Petri-plate containing a 5 mm fungal disc on PDA. Parafilm was used to seal the bottoms of the two Petri dishes. The control was prepared in the same manner but without the bacterial culture. After 6 days of incubation at 25 °C, the observations were recorded. The inhibition rate of mycelial growth was determined using the equation used for the in vitro confrontation bioassay. Each pathogen/bacterial isolate was subjected to two independent experiments with four replicates.

### 2.6. Bacterial Cell-Free Filtrate Effects on Mycelial Growth

Antibiosis was performed using the bacterial supernatant by including the filtrate of bacterial isolates to assess the role of diffusible compounds in the antifungal action [47]. An aliquot of the bacterial suspension (100 µL) at 1 × 10^8^ CFU/mL was inoculated in flasks containing nutrient broth medium (NB). After 3 days under shaking (130 rpm) at 28 °C, the cultures were centrifuged (5000 rpm) for 25 min, and then the supernatant from each isolate was filtered using a syringe with a Millipore filter (0.22 µm pore diameter). The cell-free filtrates were added into a PDA medium (45–50 °C) to obtain a concentration reaching 10% (*v*/*v*). Furthermore, the control included a liquid NB with the PDA medium only. In the Petri-plate center, a mycelial plug measuring 5 mm taken from a fresh culture was placed and incubated at 25 °C. Six days after incubation, the pathogen’s diameter was recorded and utilized to estimate the rates of inhibition as indicated above. The assay was conducted twice with 4 replicates for each bacterial isolate.

### 2.7. In Vivo Bioassay

The potential of bacteria to minimize the severity of olive root rot disease under greenhouse conditions on one-year-old plants was tested. A pure 24-hour culture of each isolate was used to prepare the bacterial inoculum. For three days, the cultures were placed, under shaking at 100 rpm, in flasks containing PBS. The resulting suspension concentration was adjusted to 1 × 10^9^ CFU/mL (OD 600 = 0.8–1) using sterile distilled water [48]. The inoculation of olive seedlings with the oomycete pathogen was done following the experimental protocol of Santilli et al. [49]. The inoculum consisted of a culture of the pathogen aged 21 days grown in the dark at 25 ± 1 °C in pots filled with a sterilized medium made of 50 mL of V8 juice with 50 g of wheat seeds. Once the inoculum was prepared, the plants were removed carefully from their substrate, cleaned from soil debris, and washed with sterile distilled water (SDW). Subsequently, 10 g of the fungal inoculum was distributed around the root system of the olive seedlings and then covered with sterile soil. Afterwards, the bacterial isolates were poured by watering the plants with 200 mL of bacterial suspension (2 × 10^8^ CFU/g). Olive plants were kept in flooded soil for 24 h [50]. The infected seedlings were maintained in a greenhouse at 25 °C. Plants were irrigated 2–3 times per week. The in vivo assay was done as described in Table 1. The trial was repeated twice over time, and plants were organized in a randomized block with 6 repetitions per bacteria. After two months, the efficacy of bacterial treatment on disease symptom reduction was evaluated. A 1–5 scale was then adopted to visually estimate the severity on plant roots [51]: 1 = healthy white roots/no disease observed; 2 = 25% root rot or seemingly healthy roots + onset of root rots, 3 = 50% root rot and early browning, 4 = 75% root rot (browning of root system), and 5 = 100% dead roots.

### 2.8. Statistical Analysis

All experiments were repeated twice over time following a completely randomized design. The Arcsine transformation was used to determine the severity of the disease. Tukey’s test was conducted for means separation at a significance level (*p* ≤ 0.05) using SPSS statistical software (version 20, IBM SPSS Statistics 20, New York, NY, USA).

## 3. Results

### 3.1. Antagonism Effect

**Figure 1:**
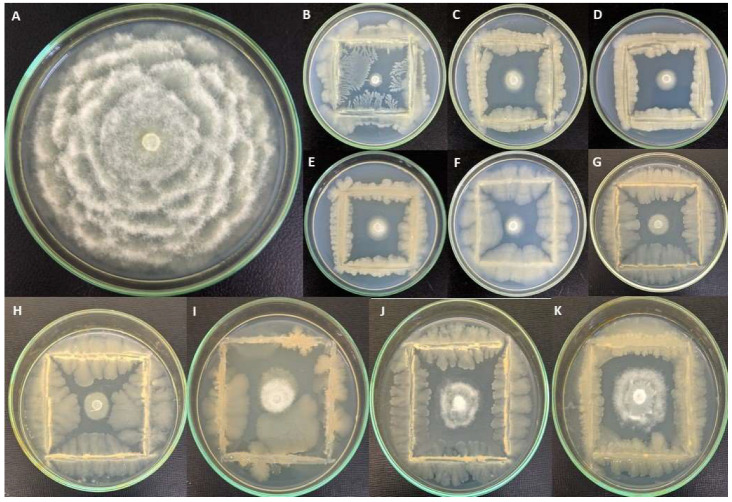
In vitro confrontation revealing antagonistic activity of bacterial strains against *P. schmitthenneri* on PDA medium after 6 days of incubation at 25 °C. (**A**) control; (**B**) ACBC1; (**C**) ACBC2; (**D**) ACBP1; (**E**) ACBP2; (**F**) SF14; (**G**) K3-7; (**H**) Bel3-4; (**I**) TG6; (**J**) BM3-5; (**K**) GH1-5.

The dual culture plate technique was applied to investigate the influence of the ten chosen bacterial isolates on the hyphal growth of *P. schmitthenneri*. The bacterial strains significantly exhibited different inhibition rates against *P. schmitthenneri* growth (Table 2 and Figure 1). *A. faecalis* ACBC1, *B. amyloliquefacienswas* SF14, and *Pantoea agglomerans* ACBC2 were significantly the most effective, with an inhibition rate of 85.15%, 81.76%, and 80.59%, respectively. The other strains presented inhibition rates ranging between 73.97% and 78.38%, with the lowest inhibition rate (60.47%) observed with *S. maltophilia* GH1-5.

### 3.2. Microscopic Observation

**Figure 2:**
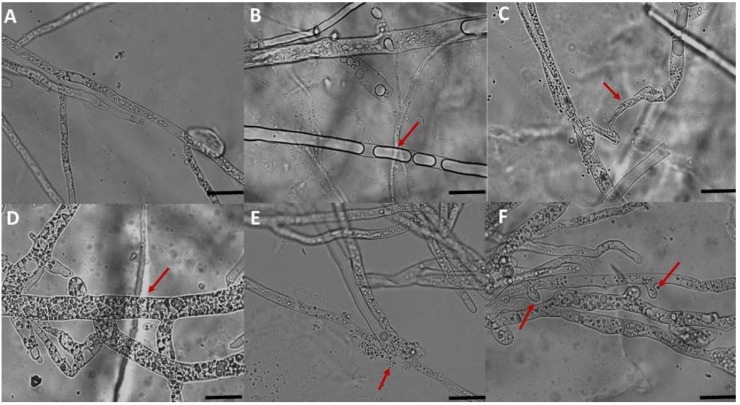
Microscopic observation (×40) of the hyphal structure of *P. schmitthenneri* co-cultured with the antagonistic bacteria after 6 days of incubation. (**A**) untreated control; (**B**) vacuolation (SF14); (**C**) deformation (ACBC2); (**D**) hyphal swelling (ACBC1); (**E**) degradation of the mycelium (BM3-5); (**F**) budding of the mycelium structure (Bel3-4). Changes in the hyphae and mycelia are indicated by arrows. Scale bar = 20 µm.

Microscopic observations of *P. schmitthenneri* mycelium co-cultured with antagonistic bacteria revealed significantly altered morphology and cytological abnormalities when compared with the untreated control (Figure 2A). Generally, the modifications corresponded to vacuolation, deformation, and hyphal swelling or budding of the mycelium structure (Figure 2B–D,F) and were infrequently linked with mycelium destruction and release of cytoplasmic contents (Figure 2E).

### 3.3. Effect of Bacterial Volatile Organic Compounds on Mycelial Growth

**Figure 3:**
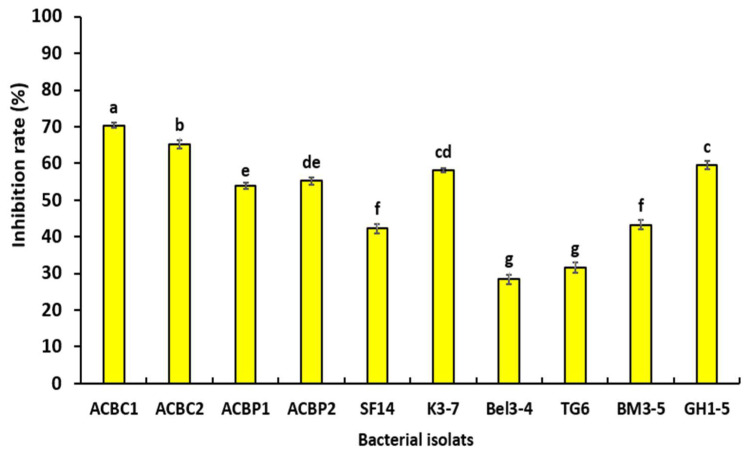
Effect of volatile organic compounds (VOCs), which were produced by tested bacteria on the inhibition of hyphal growth of *P. schmitthenneri* after 6 days of incubation at 25 ± 1 °C. Data in the figure are the average of two separate experiments with four replicates. According to the Tukey test, values with the same letter were not significantly different (*p* ≤ 0.05).

Statistical analysis demonstrated that bacterial VOCs influenced the mycelial development of *P. schmitthenneri,* after 6 days of incubation at 25 ± 1 °C, in comparison with the growth of the control (pathogen only) (Figure 3). The antifungal activity of bacterial VOCs ranged from a maximum of 70.32% (ACBC1) to a minimum of 28.37% (Bel3-4). In addition, results showed that six tested isolates gave an inhibition rate higher than 50% (ACBC1, ACBC2, GH1-5, K3-1, ACBP2, and ACBP1).

### 3.4. Effect of Bacterial Filtrates on Mycelial Growth

**Figure 4:**
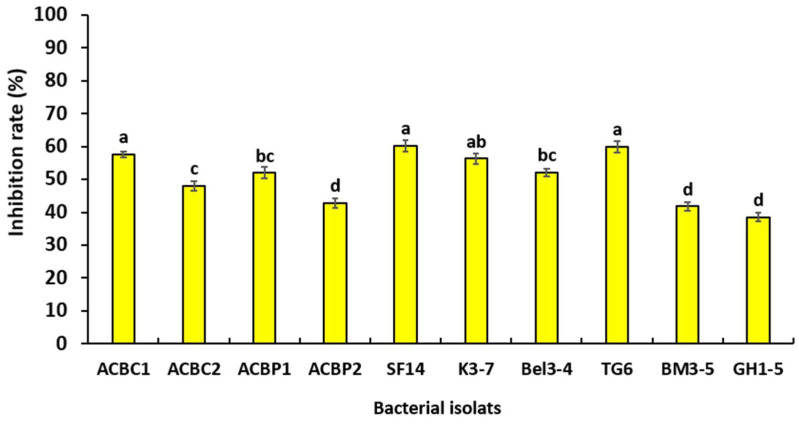
Effect of bacterial cell-free filtrates 10% *v*/*v* on the inhibition of mycelial growth of *P. schmitthenneri* after 6 days of incubation. Data in the figure represent the mean of two independent trials with 4 replicates. Treatments with the same letter were not significantly different according to the Tukey test (*p* ≤ 0.05).

Statistical analysis showed that there was a significant difference between the inhibition rates of mycelial growth obtained with cell-free bacterial filtrates used against *P. schmitthenneri* at *p* ≤ 0.05 (Figure 4). The cell-free filtrates of the bacterial isolates SF14, TG6, and ACBC1 have the highest reduction of mycelial growth with inhibition rates reaching 60.16%, 59.96%, and 57.56%, respectively.

### 3.5. In Planta Bioassay

**Figure 5:**
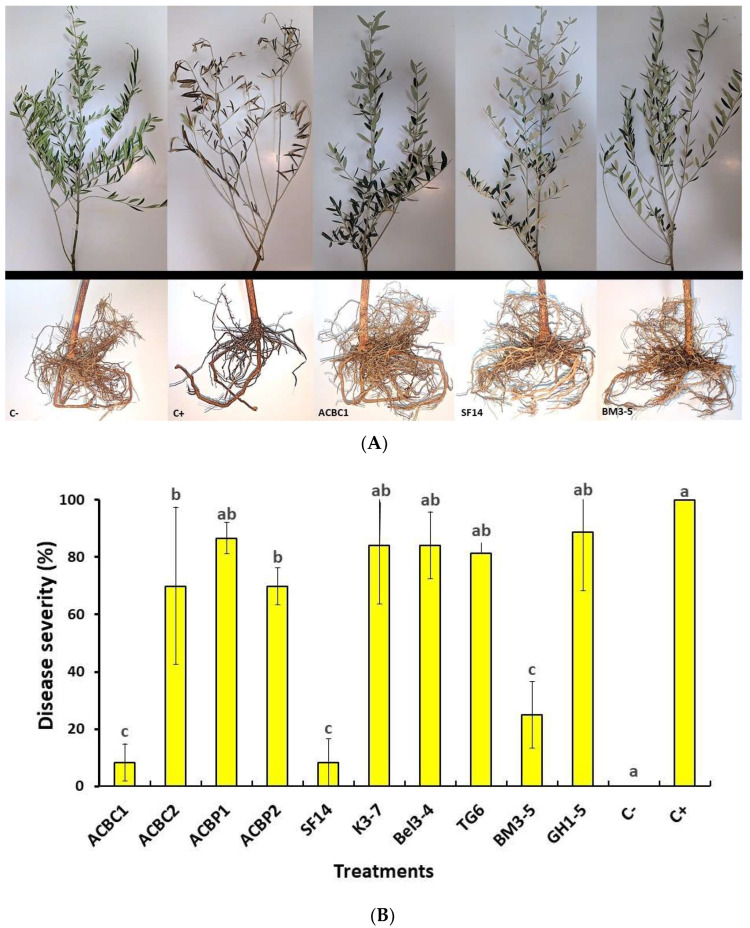
(**A**) Symptoms of olive root rot disease on a one-year-old plant treated with bacterial isolates ACBC1, SF14, and BM3-5 and inoculated with *P. schmitthenneri* after two months within glasshouse conditions. C+: positive control (olive plants inoculated only with the pathogen), and C−: negative control (plants treated only with water in the absence of the pathogen). (**B**) Observation of disease severity (%) on the root of olive trees treated with bacterial suspension of the ten isolates (2 × 10 ^8^ CFU/mL) and inoculated with *P. schmitthenneri*, after 2 months of incubation at 25 °C within greenhouse conditions. C+, positive control (pathogen only), and C−, negative control (plants treated only with water in the absence of the pathogen). Histograms represent the mean value of disease severity of two trials over time with six replicates. Error bars represent standard error, and values of plant severity with the same letter were not significantly different according to the Tukey test (*p ≤* 0.05).

The ten bacterial strains were evaluated in the greenhouse based on their in vitro findings to validate their inhibitory effects and abilities to control olive root rot disease. Statistical analysis showed that there was a significant difference between the ten strains at *p* ≤ 0.05. The strains ACBC1, SF14, and BM3-5 were particularly successful in reducing root rot disease after two months of post-incubation, with disease severity reaching 8.33%, 8.33%, and 25%, respectively (Figure 5A,B). These bacterial isolates showed results that were quite comparable to the negative control (without *P. schmitthenneri*). However, the remaining bacteria were demonstrated to be less efficient to control *P. schmitthenneri* infection (Figure 5B).

## 4. Discussion

The use of microorganisms is a safe and viable alternative to synthetic fungicides for the control of soil-borne diseases. This approach requires searching for possible BCA candidates with strong antagonistic properties. In this sense, the present study evaluated the capacity of ten bacterial isolates, previously characterized and selected for their antagonistic activity against wide pathogens [38,39,40], to control olive root rot disease caused by *P. schmitthenneri*.

Our findings showed that bacterial strains have a significant in vitro antagonistic capacity on mycelial growth ranging from 60.47 to 85.15%. The highest inhibition rates were observed in treatments with the antagonistic isolates ACBC1 followed by SF14 and then ACBC2. Previous investigations have also shown the effectiveness of antagonistic bacteria in the management of *Pythium* spp. [52,53,54,55,56]. In accordance with our results, Lahlali et al. [38] reported the antagonistic activity of two strains, namely, *Alcaligenes faecalis* ACBC1 and *Bacillus amyloliquefaciens* SF14, in a dual culture test with inhibition rates of 96.3% and 91.9%, respectively, against *Monilinia fructigena*. Likewise, Bardin et al. [57] found that the *Pantoea agglomerans* 2-2 strain was successful in reducing *Pythium* sp. mycelial development in vitro, which was related to its capacity to secrete extracellular protease. In addition, the *P. agglomerans* ENA1 strain showed a very significant antagonistic activity against *Macrophomina phaseolina*, with an inhibition rate reaching 89% compared with the control [58]. *Stenotrophomonas maltophilia* isolate W81, a multidrug-resistant bacteria, has also shown an ability to control *P. ultimum* through the production of lytic enzymes including protease, pectinase, and chitinase and the secretion of VOCs [59]. The significant antagonistic potential of other bacteria has been extensively studied such as *S. maltophilia* CR71 against *Colletotrichum nymphaea* [60]; *B. xiamenensis* PM14 against *P. splendens* [61]; *B. subtilis* DCl1 against *P. myriotylum*, *Phytophthora infestans*, and *Rhizoctonia solani* [62]; and *B. subtilis* CU12 against *Alternaria solani*, *P. sulcatum*, *Botrytis cinerea*, and *F. sambucinum* [63]; among others.

In this study, both *S. multivorum* and other antagonists reduced the mycelial development of *P. schmitthenneri* but with varying degrees of inhibition related specifically to the mode of action of each isolate. Three tests were conducted in vitro to study the modes of action of selected bacterial isolates. Under a microscope, the results revealed an alteration of mycelial structure in the form of deformation, vacuolation, and swelling of *P. schmitthenneri* mycelium co-cultured with each isolate. These cytological changes can be due to substances synthesized by the bacteria [64]. In the same line, Cheffi et al. [65] examined the potential of *B. velezensis* OEE1 to control the strain Fso1 of *F. solani*, finding substantial cytoplasm vacuolization and mycelial lysis.

The mode of action occurs generally via the production of antifungal substances and VOCs [66,67,68], or parasitism through the secretion of lytic enzymes and lipopeptides [69,70,71]. The production of VOCs has been widely implicated in the bio-control of soil-borne fungi [72,73,74]. For instance, Wang et al. [75] found that *B. halotolerans* KLBC XJ-5 highly inhibited the hyphal growth of *B. cinerea* after five days of incubation, with an inhibition rate higher than 73.7%. In that study, the inhibition was correlated with the secretion of lipopeptides. In addition, the isolate *B. halotolerans* BFOA1/BFOA4 produced a variety of secondary metabolites and successfully controlled *B. cinerea*, *F. oxysporum* f. sp. *Albedinis*, *P. infestans*, *R. bataticola*, and *A. alternata* [76].

In our study, the inhibitory rate of bacterial filtrates ranged from 38.49% to 60.16%, suggesting that the ten isolates used might be sources of diverse secondary metabolites. According to Li et al. [47], the inhibition rates of the bacterial cell-free filtrates are associated with their increasing concentration; the inhibition becomes greater as the filtrate concentrations increase. Extracellular metabolites from *B. amyloliquefaciens* strain QSB-6 significantly affected *Fusarium* mycelial development and spore germination [77].

The emission of VOCs has been extensively linked to the biological control of soil-borne pathogens [72,73,74]. The findings of the indirect confrontation in this study showed that the ten strains could generate VOCs and limit the mycelial growth of *P. schmitthenneri*. According to Sànchez-Fernàndez et al. [78], *Nodulisporium* sp. GS4d2II1a reduced the hyphal development of *P. aphanidermatum* and other pathogenic fungi by emitting VOCs. These VOCs are a mixture of small volatile compounds that are present in a gaseous state under ambient temperatures (i.e., 1 atm pressure and 25 °C temperature) due to their low water solubility and high vapor pressure [74,79].

In this work, an in vivo bioassay on the root of olive trees was assessed to validate the antagonistic capacity of the bacterial isolates in field conditions. Tree isolates of *A. faecalis* ACBC1, *B. amyloliquefaciens* SF14, and *B. halotolerans* BM3-5 showed a very significant capacity to decrease the disease severity of root rot. In a greenhouse environment, the strain *B. amyloliquefaciens* Y1 lowered the incidence of *Fusarium* wilt disease in tomatoes when compared to the control [80]. Furthermore, this isolate has been effective in the biocontrol of several other pathogens, including *Phytophthora capsici*, *Rhizoctonia solani*, and *Botrytis cinerea*. Taken together, the antagonistic strains *A. faecalis* ACBC1 and *B. amyloliquefaciens* SF14 were the most successful in suppressing the pathogen *P. schmithenneri* in both in vitro and in planta experiments.

Numerous research studies have shown that antagonistic bacteria constitute a major source of lytic enzymes, which are utilized to limit the spread of fungal pathogens and diminish their pathogenicity [81,82]. Our antagonistic bacteria were previously characterized based on their capacity to synthesize lytic enzymes, and all of them could produce at least two lytic enzymes (amylase, cellulose, and protease) (Table S1) [38,39].

The mycelia of *P. debaryanum*, in this study, were entirely lysed in many regions. In the same context, Salem and Abdel-Rahman et al. [83] reported that *B. subtilis* MK537378 and *Trichoderma reesei* MK934489 showed a high capacity for cellulase enzyme production as well as antagonistic activity against *P. debaryanum*. Aydi Ben Abdallah et al. [84] also revealed that *A. faecalis* S18 and *B. cereus* S42 produced protease and chitinase and concluded that the suppression of mycelial growth of *F. oxysporm* f. sp. *lycopersici* by these two antagonistic bacteria was due to the synthesis of cell wall destroying enzymes. Moreover, Sen et al. [85] found that the strain SSB17 of *A. faecalis* might produce the α-amylase. In addition, the antagonistic activity of four *Trichoderma* strains against *P. myriotylum* was correlated with the production of protease, cellulase, and xylanase [37].

Furthermore, hydrogen cyanide (HCN) is a volatile compound biosynthesized from glycine using HCN synthase [86]. Our results indicated that only *P. agglomerans* ACBP1 was able to produce HCN. The latter was reported in various studies to suppress soil-borne pathogens [87,88,89]. Indeed, HCN acts by inhibiting cytochrome oxidase in the electron transport chain and energy supply to cells, leading to the death of phytopathogenic fungi [90].

In this study, the tested antagonistic bacteria exhibit other features. Amongst them, genes implicated in lipopeptide secretion and that are important in antibiosis were also detected in the isolates (Table S1) [38,39]. These antimicrobial compounds can suppress fungal growth, particularly fengycin, which has a potent antifungal activity toward filamentous fungi [91,92]. *Bacillus* spp. has been shown to produce a wide range of lipopeptides (e.g., surfactins, iturins, and fengycins) [93,94,95,96,97]. According to Zhang et al. [98], genes coding for lipopeptide biosynthesis have been found in most tested *Bacillus* isolates. Lee et al. [99] suggested that the inhibitory effect of *B. amyloliquefaciens* DA12 was related to iturin A and volatile heptanones production, making it a good BCA candidate against *Fusarium* diseases.

## 5. Conclusions

The effectiveness of antagonistic bacteria in controlling the olive root rot disease caused by *P. schmitthenneri* was investigated in this research. The three in vitro bioassays revealed a significant effect of the ten bacterial strains on the inhibition of the mycelial growth of the pathogen. Under greenhouse conditions, two bacteria, namely, *Alcaligenes faecalis* ACBC1 and *Bacillus amyloliquefaciens* SF14, showed promising results as they were highly effective in controlling the disease severity. The ability of these BCAs to produce lytic enzymes and lipopeptides determined their effectiveness. These results provide new control alternatives for the establishment of biocontrol strategies to manage sustainably the olive root rot disease. Therefore, two bacterial isolates, ACBC1 and SF14, were proposed to be used to control and prevent disease damage. However, additional experiments under natural conditions are needed to confirm their large-scale biocontrol potential before going on to the next stage of formulation of these antagonistic bacterial isolates as commercial bio-fungicide products.

## Figures and Tables

**Table 1:** Treatments used in the in vivo bioassays.

Treatments	Composition	Repetitions	Period
ACBC1	SS + pathogen + ACBC1	6	2 months
ACBC2	SS + pathogen + ACBC2		
ACBP1	SS + pathogen + ACBP1		
ACBP2	SS + pathogen + ACBP2		
SF14	SS + pathogen + SF14		
K3-7	SS + pathogen + K3-7		
Bel3-4	SS + pathogen + Bel3-4		
TG6	SS + pathogen + TG6		
BM3-5	SS + pathogen + BM3-5		
GH1-5	SS + pathogen + GH1-5		
C+	SS + Pathogen		
C−	SS alone		

SS: sterilized soil.

**Table 2:** The in vitro inhibition rates (%) of the *P. schmitthenneri* mycelial growth after 6 days of incubation at 25 °C in darkness.

Bacterial Isolate Code	Species	Accession Numbers	Inhibition Rates (%)
ACBC1	*Alcaligenes faecalis*	KY357285	85.14 ± 1.13 ^a^
ACBC2	*Pantoea agglomerans*	KY357286	80.58 ± 1.31 ^bc^
ACBP1	*P. agglomerans*	KY357287	77.79 ± 0.64 ^cde^
ACBP2	*P. agglomerans*	KY357288	78.38 ± 0.64 ^bcd^
SF14	*Bacillus amyloliquefaciens*	KY357298	81.76 ± 1.76 ^ab^
K3-7	*B. xiamenensis*	MW843010	77.89 ± 1.96 ^cd^
Bel3-4	*Sphingobacterium multivorum*	MW856827	73.97 ± 0.87 ^f^
TG6	*B. subtilis*	MW847628	74.45 ± 1.48 ^ef^
BM3-5	*B. halotolerans*	MW847951	76.49 ± 0.53 ^def^
GH1-5	*Stenotrophomonas maltophilia*	MW848819	60.47 ± 1.04 ^g^

Data represent mean ± standard deviation (SD) of two trials over time with three replicates. Inhibition rates with the same letter are not significantly different according to the Tukey test performed on mycelial growth (*p* ≤ 0.05).

## Data Availability

The data that support the findings of this study are available from the corresponding author upon reasonable request.

## Supplementary Materials

The following supporting information can be downloaded at: www.mdpi.com/article/10.3390/microorganisms10081635/s1, Table S1: Summary of relative hydrolytic enzyme and lipopeptides exhibited by the ten bacteria [38,39].
