# Supplementary material for: Potential for Biological Control of Pythium schmitthenneri Root Rot Disease of Olive Trees (Olea europaea L.) by Antagonistic Bacteria
Source: Microorganisms. 2022 Aug 12;10(8):1635. doi: 10.3390/microorganisms10081635 (PMC9412840; doi:10.3390/microorganisms10081635)
Supplement: Supplementary file 1 [file microorganisms-10-01635-s001.zip › microorganisms-1836305-supplementary.pdf]

Supplementary Table S1: Summary of relative hydrolytic enzyme and lipopeptides exhibited by the ten bacterial isolates [38,39].

Isolate	Amylase	Protease	Cellulase	HCN	Bacillomycin	Fengycin	Iturin	Surfactin
**ACBC1**	+	+	+	-	-	-	+	-
**ACBC2**	-	+	-	-	+	+	-	-
**ACBP1**	+	-	+	+	-	-	+	-
**ACBP2**	+	+	-	-	-	-	-	-
**SF14**	-	+	+	-	+	+	-	+
**K3-7**	+	+	+	-	-	-	-	-
**Bel3-4**	+	+	+	-	+	-	-	-
**TG6**	+	+	+	-	+	-	-	-
**BM3-5**	+	+	+	-	-	+	+	-
**GH1-5**	+	+	+	-	+	+	-	-
